# Alcoholics Anonymous: Who Benefits?

**Published:** 1994

**Authors:** J. Scott Tonigan, Susanne HillerSturmhöfel

**Affiliations:** J. Scott Tonigan, Ph.D., is deputy director of the Research Division at the Center on Alcoholism, Substance Abuse, and Addictions; University of New Mexico; Albuquerque, New Mexico. Susanne Hiller-Sturmhöfel, Ph.D., is an associate editor of Alcohol Health & Research World

Alcoholics Anonymous (AA) is the most popular self-help organization for individuals with alcohol-related problems. This includes both alcohol-dependent and, to a lesser extent, alcohol-abusing drinkers. For many people, self-help through AA is the only alcoholism treatment they receive. Other members join the fellowship before entering professional treatment or are introduced to AA as a component of their professional treatment. Attendance at AA also commonly is recommended as after-care following professional treatment. Yet experience shows that not all clients benefit from AA to the same extent. Therefore, two questions arise: Who does well in AA, and why do these people succeed?

These questions do not have simple answers, however, because outcome (i.e., reduction of drinking or improvement of psychological and social characteristics) associated with AA, as with any kind of alcoholism treatment, is influenced by many characteristics of the clients and the AA groups. For example, the success of AA participation depends not only on an individual’s initial decision to attend AA but also on the degree of his or her involvement in AA (e.g., frequency of attendance at meetings, “sharing” at meetings, or serving as or having an AA sponsor). Even similar levels of AA involvement may result in different outcomes for different people, depending on the individual’s characteristics and experiences with professional treatment.

Despite four decades of AA research, no clear picture has emerged as to which patient characteristics can predict a positive outcome with AA and, therefore, can be used as criteria for matching patients to AA. This is due in part to the limitations and variability of methodological approaches used in the studies. Most investigators recruit their samples from patients in inpatient or outpatient treatment settings. Some studies retrospectively analyze patients with previous AA experience to identify personal characteristics that predicted AA involvement. In other studies, patients are monitored after professional treatment to determine which characteristics may motivate them to join AA and how AA affiliation influences outcome. In both approaches, the kind and impact of the professional treatment often is ignored. Other confounding factors in research about AA include an incomplete understanding of processes within AA and differences among various AA groups.

To date, only three randomized clinical trials have examined the efficacy of AA participation, either with or without additional simultaneous treatment approaches (Ditman et al. 1967; Brandsma et al. 1980; Walsh et al. 1991). The vast majority of AA studies, however, have focused on two narrower questions: Which factors predict whether a person will join AA? And how does involvement in AA predict outcome? In an attempt to answer these two questions, Emrick and colleagues (1993) reviewed 107 previously published AA studies. Although their analysis provided estimates of the magnitude of the relationships determining AA affiliation and drinking outcome, it also acknowledged that many relationships may differ when study findings are grouped by client characteristics. Tonigan and colleagues (1994) extended the initial analyses by taking into account factors such as sample gender and origin (i.e., inpatient versus outpatient). This article integrates the findings of these two reviews and concludes with recommendations for future research of AA.

## Who Joins AA?

To determine which drinkers were most likely to join AA, Emrick and colleagues (1993) reviewed 33 studies^1^ that addressed this question, analyzing 31 demographic and drinking-related client characteristics. The characteristic most strongly correlated with joining AA was the drinkers’ previous use of external support mechanisms to stop drinking. The drinkers’ demographic characteristics, such as gender, age, and education, were not related to whether or not they joined AA. Factors related to alcohol consumption, such as quantity consumed daily, obsessive preoccupation with alcohol, severity of physical dependence, and loss of control while drinking, however, had some correlational value. For example, drinkers who had higher levels of alcohol consumption had a greater likelihood of attending AA.

Tonigan and colleagues (1994) analyzed whether sample origin (i.e., sample recruitment from outpatient or inpatient settings) affected the correlation between consumption-related factors and AA affiliation. The study found that although the overall rate of AA affiliation was comparable for outpatient and inpatient samples, affiliation was modestly correlated to consumption-related factors only in outpatient samples—no such correlation existed in inpatient samples. One explanation for this difference could be that, in general, there was much greater variation in these factors (e.g., alcohol consumption levels of the patients) among inpatient samples than among outpatient samples. Such variation could attenuate the relationship between consumption-related factors and AA affiliation.

## Does AA Involvement Reduce Drinking?

Without taking into consideration patients’ professional treatment experiences, Emrick and colleagues (1993) reviewed 16 studies^1^ to determine whether the extent of AA involvement predicted treatment outcome. Most of the studies found that greater AA involvement could modestly predict reduced alcohol consumption.

When Tonigan and colleagues (1994) examined the influence of gender on this correlation, they found that the relationship between AA involvement and abstinence was stronger in studies that analyzed only men than in studies that included men and women. This finding indicates that men and women may respond differently to AA and that AA involvement may be less beneficial to women. One potential explanation is that women may require different treatment settings than men for optimal treatment outcome (Beckman 1994). Some studies indicate that women may prefer more one-on-one treatment (Jarvis 1992) and, consequently, may benefit less from the group-oriented AA setting. Alternatively, AA involvement may be less beneficial for women because co-occurring disorders that are more prevalent among women, such as depression, often are not addressed explicitly in AA programs. This theory is supported by studies^1^ that analyzed alcohol consumption and AA attendance in clients who already had completed professional treatment, during which any coexisting psychiatric disorders presumably would have been addressed. Therefore, the women would not have to rely on AA to serve as their sole source of treatment for both alcohol-related and psychiatric problems. These studies found only small differences between men’s and women’s outcome as a result of AA involvement.

Other studies^1^ analyzed the relationship between AA involvement and improved psychosocial functioning. These studies used measures such as marital satisfaction; employment status; or scores on the Minnesota Multiphasic Personality Inventory, a questionnaire used to measure psychological functioning. Tonigan and colleagues (1994) found modest positive relationships between AA attendance and improvement of these measures. However, psychosocial improvement was not the same for all client populations. For example, among clients who received no professional treatment, men appeared to improve more than women. Among clients receiving professional treatment in addition to participating in AA, those in outpatient programs reported greater psychological improvement as a result of AA attendance than did those in inpatient programs.

## Recommendations for Future AA Research

Although the analysis of AA studies suggests some patient characteristics that influence affiliation with AA or drinking and psychological outcome, the existing research still has severe methodological flaws, as was mentioned earlier. For example, the patient samples used in many studies do not represent adequately the general AA member population, and demographic patient characteristics often are not described thoroughly. Also, the instruments used to measure drinking, AA affiliation and involvement, and outcome often rely on patients’ self-reporting, a method that inherently involves variability and may lack reliability. A plethora of innovative research approaches and questions have been suggested to strengthen AA research (McCrady and Miller 1993), such as those discussed below.

First, patient samples in AA studies should represent AA member composition more accurately. In particular, the under-representation of adolescents and women in AA research must be corrected. To be more informative, studies also should report routinely patient characteristics, such as age, gender, marital and employment status, and severity of drinking problems.

Second, followup protocols for AA studies should be extended. With some exceptions (e.g., Sheeren 1988; Vaillant 1983), AA studies have not conducted long-term followup. In the studies reviewed by Emrick and colleagues (1993), the average assessment time after affiliation with AA was 18 weeks. Given the lifelong commitment expected of AA members, it is doubtful whether such a short period is sufficient to detect meaningful changes.

Third, factors promoting AA involvement must be better identified and understood. Evidence suggests that the extent of involvement in AA, rather than the frequency of attendance, predicts how individuals fare in AA (e.g., Snow et al. 1994). However, there still is no consensus on how to assess involvement and even less consensus on the factors that influence whether, and how much, a person becomes involved. Health care professionals and researchers, because of their clinical experience and contact with AA members, could be valuable resources for developing reliable instruments to measure involvement.

Fourth, future research should pay more attention to patient-treatment matching approaches and examine how different types of professional alcoholism treatment and different patient characteristics relate to AA involvement and drinking behavior. For example, existing evidence suggests that women do better in AA after having had prior professional treatment, rather than without having had such treatment, and that AA members who receive outpatient treatment fare better than those who receive inpatient treatment.

A patient-treatment matching approach also could include comparisons of the philosophies behind different professional treatment approaches and AA. Philosophical similarity between a specific program and AA may increase a patient’s acceptance of AA principles, thus improving the patient’s involvement and, consequently, outcome with AA. Conversely, philosophical differences could negatively affect a patient’s involvement and outcome with AA.

When matching clients to AA, differences between individual AA groups also may need to be considered. AA is not a single entity. A study by Montgomery and colleagues (1993) found that AA groups vary in their social structure and their characteristics, such as perceived cohesiveness, aggressiveness, and expressiveness. Some clients may be more attracted and responsive to specific group characteristics than others. Consequently, it may not be realistic to expect to find general predictors of affiliation and outcome with AA.

## Footnotes

1 The complete list of references for these studies is available from the first author.

## References

[b1-arhw-18-4-308] Beckman LJ (1994). Treatment needs of women with alcohol problems. Alcohol Health & Research World.

[b2-arhw-18-4-308] Brandsma JM, Maultsby MC, Welsh RJ (1980). Outpatient Treatment of Alcoholism: A Review and Comparative Study.

[b3-arhw-18-4-308] Ditman KS, Crawford GG, Forgy EW, Moskowitz H, MacAndrew C (1967). A controlled experiment on the use of court probation for drunk arrests. American Journal of Psychiatry.

[b4-arhw-18-4-308] Emrick CD, Tonigan JS, Montgomery H, Little L, McCrady BS, Miller WR (1993). Alcoholics Anonymous: What is currently known?. Research on Alcoholics Anonymous: Opportunities and Alternatives.

[b5-arhw-18-4-308] Jarvis TJ (1992). Implications of gender for alcohol treatment research: A quantitative and qualitative review. British Journal of Addiction.

[b6-arhw-18-4-308] McCrady BS, Miller WR (1993). Research on Alcoholics Anonymous: Opportunities and Alternatives.

[b7-arhw-18-4-308] Montgomery HA, Miller WR, Tonigan JS (1993). Differences among AA groups: Implications for research. Journal of Studies on Alcohol.

[b8-arhw-18-4-308] Sheeren M (1988). The relationship between relapse and involvement in Alcoholics Anonymous. Journal of Studies on Alcohol.

[b9-arhw-18-4-308] Snow MG, Prochaska JO, Rossi JS (1994). Processes of change in Alcoholics Anonymous: Maintenance factors in long term sobriety. Journal of Studies on Alcohol.

[b10-arhw-18-4-308] Tonigan JS, Toscova R, Miller WR Meta-Analysis of the Alcoholics Anonymous Literature: A Search for Moderating Variables.

[b11-arhw-18-4-308] Vaillant GE (1983). The Natural History of Alcoholism: Causes, Patterns, and Paths to Recovery.

[b12-arhw-18-4-308] Walsh DC, Hingson RW, Merrigan DM, Morelock Levenson S, Cupples LA, Heeren T, Coffman GA, Becker CA, Barker TA, Hamilton SK, McGuire TG, Kelly CA (1991). A randomized trial of treatment options for alcohol-abusing workers. New England Journal of Medicine.

